# Supplementary material for: Evaluation of general anesthesia protocols for a highly controlled cardiac ischemia-reperfusion model in mice
Source: PLoS One. 2024 Oct 25;19(10):e0309799. doi: 10.1371/journal.pone.0309799 (PMC11508169; doi:10.1371/journal.pone.0309799)
Supplement: S1 Fig — (PDF) [file pone.0309799.s001.pdf]

id mouse	protocol	Loss of righting reflex	Induction	Surgical Stage	Recovery	Immobilization	Return ofrighting reflex
KX-1	Protocol 1 : Ket/Xyl	2	53	5	35	93	95
KX-2		2	18	45	25	88	90
KX-3		2	28	5	25	58	60
KX-4		1	34	45	10	89	90
KX-5		3	22	80	40	142	145
KX-6		2	33	5	65	103	105
KX-7		3	27	10	95	132	135
KX-8		2	23	55	55	133	135
KX-9		1	34	30	45	109	110
KX-10		2	0	0	0	73	75
KX-11		3	62	5	40	107	110
KX-12		2	0	0	0	78	80
Median		2.0	27.5	7.5	37.5	98.0	100.0
Q1		2.0	19.0	5.0	13.8	80.5	82.5
Q3		2.8	34.0	45.0	52.5	126.3	128.8
Interquartille		0.8	15.0	40.0	38.8	45.8	46.3
AX-1	Protocol 2 : Alf/Xyl	2	18	25	50	93	95
AX-2		2	0	0	0	83	85
AX-3		2	0	0	0	63	65
AX-4		3	0	0	0	67	70
AX-5		2	33	5	35	73	75
AX-6		2	13	55	15	83	85
AX-7		2	28	40	10	78	80
AX-8		2	0	0	0	58	60
AX-9		2	13	15	50	78	80
AX-10		2	18	10	45	73	75
AX-11		1	19	40	20	79	80
AX-12		2	0	0	0	78	80
Median		2.0	13.0	7.5	12.5	78.0	80.0
Q1		2.0	0.0	0.0	0.0	68.5	71.3
Q3		2.0	18.8	36.3	42.5	82.0	83.8
Interquartille		0.0	18.8	36.3	42.5	13.5	12.5
KM-1	Protocol 3 : Ket/Med	1	39	80	30	149	150
KM-2		1	44	75	25	144	145
KM-3		1	19	120	30	169	170
KM-4		1	24	115	50	189	190
KM-5		1	19	140	20	179	180
KM-6		1	19	120	45	184	185
KM-7		1	19	120	40	179	180
KM-8		1	14	125	40	179	180
KM-9		2	23	115	35	173	175
KM-10		1	19	95	30	144	145
KM-11		1	9	125	35	169	170
KM-12		1	24	125	40	189	190
Median		1.0	19.0	120.0	35.0	176.0	177.5
Q1		1.0	19.0	100.0	30.0	154.0	155.0
Q3		1.0	24.0	125.0	40.0	182.8	183.8
Interquartille		0.0	5.0	25.0	10.0	28.8	28.8
AM-1	Protocol 4 : Alf/Med	1	19	25	50	94	95
AM-2		2	13	40	45	98	100
AM-3		2	13	110	5	128	130
AM-4		2	18	60	60	138	140
AM-5		2	23	105	15	143	145
AM-6		2	23	45	25	93	95
AM-7		2	28	25	55	108	110
AM-8		2	13	105	15	133	135
AM-9		2	18	75	50	143	145
AM-10		2	28	85	30	143	145
AM-11		2	33	20	50	103	105
AM-12		2	38	5	60	103	105
Median		2.0	21.0	52.5	47.5	118.0	120.0
Q1		2.0	14.3	25.0	17.5	99.3	101.3
Q3		2.0	28.0	100.0	53.8	141.8	143.8
Interquartille		0.0	13.8	75.0	36.3	42.5	42.5
KMi-1	Protocol 5 : Ket/Mid	1	14	30	25	69	70
KMi-2		1	9	35	35	79	80
KMi-3		1	19	30	15	64	65
KMi-4		1	14	35	10	59	60
KMi-5		1	9	25	20	54	55
KMi-6		1	14	25	30	69	70
KMi-7		1	9	35	30	74	75
KMi-8		1	9	55	30	94	95
KMi-9		1	9	20	45	74	75
KMi-10		1	19	25	35	79	80
KMi-11		1	14	30	40	84	85
KMi-12		1	9	40	35	84	85
Median		1.0	11.5	30.0	30.0	74.0	75.0
Q1		1.0	9.0	25.0	21.3	65.3	66.3
Q3		1.0	14.0	35.0	35.0	82.8	83.8
Interquartille		0.0	5.0	10.0	13.8	17.5	17.5
