# Supplementary material for: Evaluation of general anesthesia protocols for a highly controlled cardiac ischemia-reperfusion model in mice
Source: PLoS One. 2024 Oct 25;19(10):e0309799. doi: 10.1371/journal.pone.0309799 (PMC11508169; doi:10.1371/journal.pone.0309799)
Supplement: S2 Fig — (PDF) [file pone.0309799.s002.pdf]

id mice	Protocol	Loss of righting reflex	Induction	Surgery stage	recovery	Immobilization	return of righting reflex
KM-1	**Ket/Med (Butor)**	1	39	80	30	149	150
KM-2		1	44	75	25	144	145
KM-3		1	19	120	30	169	170
KM-4		1	24	115	50	189	190
KM-5		1	19	140	20	179	180
KM-6		1	19	120	45	184	185
KM-7		1	19	120	40	179	180
KM-8		1	14	125	40	179	180
KM-9		2	23	115	35	173	175
KM-10		1	19	95	30	144	145
KM-11		1	9	125	35	169	170
KM-12		1	24	125	40	189	190
Median		1.0	19.0	120.0	35.0	176.0	177.5
Q1		1.0	19.0	100.0	30.0	154.0	155.0
Q3		1.0	24.0	125.0	40.0	182.8	183.8
Interquartille		0.0	5.0	25.0	10.0	28.8	28.8
KMBupre-1	**Ket/Med (Bupre)**	1	14	140	15	169	170
KMBupre-2		1	14	135	15	164	165
KMBupre-3		1	19	120	25	164	165
KMBupre-4		1	19	115	20	154	155
KMBupre-5		1	14	120	15	149	150
KMBupre-6		1	14	115	10	139	140
KMBupre-7		1	19	130	25	174	175
KMBupre-8		1	19	130	20	169	170
KMBupre-9		1	19	90	25	134	135
KMBupre-10		1	19	120	25	164	165
KMBupre-11		1	14	135	10	159	160
KMBupre-12		1	14	135	40	189	190
Median		1.0	16.5	125.0	20.0	164.0	165.0
Q1		1.0	14.0	116.3	15.0	150.3	151.3
Q3		1.0	19.0	135.0	25.0	169.0	170.0
Interquartille		0.0	5.0	18.8	10.0	18.8	18.8

id mice	Protocol	Loss of righting reflex	Induction	Surgery stage	recovery	Immobilization	return of righting reflex
AM-1	**Alf/Med (Butor)**	1	19	25	50	94	95
AM-2		2	13	40	45	98	100
AM-3		2	13	110	5	128	130
AM-4		2	18	60	60	138	140
AM-5		2	23	105	15	143	145
AM-6		2	23	45	25	93	95
AM-7		2	28	25	55	108	110
AM-8		2	13	105	15	133	135
AM-9		2	18	75	50	143	145
AM-10		2	28	85	30	143	145
AM-11		2	33	20	50	103	105
AM-12		2	38	5	60	103	105
Median		2.0	21.0	52.5	47.5	118.0	120.0
Q1		2.0	14.3	25.0	17.5	99.3	101.3
Q3		2.0	28.0	100.0	53.8	141.8	143.8
Interquartille		0.0	13.8	75.0	36.3	42.5	42.5
AMBupre-1	**Alf/Med (Bupre)**	2	8	90	20	118	120
AMBupre-2		2	8	80	40	128	130
AMBupre-3		2	8	80	40	128	130
AMBupre-4		2	8	80	90	178	180
AMBupre-5		2	9	90	40	139	140
AMBupre-6		2	8	70	70	148	150
AMBupre-7		2	8	80	80	168	170
AMBupre-8		2	9	70	60	139	140
AMBupre-9		2	9	100	40	149	150
AMBupre-10		2	9	90	50	149	150
AMBupre-11		2	9	70	60	139	140
AMBupre-12		2	9	70	70	149	150
Median		1.6	8.4	80.0	55.0	143.3	145.0
Q1		1.5	8.0	70.0	40.0	130.6	132.5
Q3		2.0	8.5	90.0	70.0	148.5	150.0
Interquartille		0.5	0.5	20.0	30.0	17.9	17.5
