# Supplementary material for: Evaluation of general anesthesia protocols for a highly controlled cardiac ischemia-reperfusion model in mice
Source: PLoS One. 2024 Oct 25;19(10):e0309799. doi: 10.1371/journal.pone.0309799 (PMC11508169; doi:10.1371/journal.pone.0309799)
Supplement: S3 Fig — (PDF) [file pone.0309799.s003.pdf]

With no surgery						
id souris :	Loss of righting reflex	Induction	Surgery stage	recovery	Immobilization	return of righting reflex
AMBupre-1	2	8	90	20	118	120
AMBupre-2	2	8	80	40	128	130
AMBupre-3	2	8	80	40	128	130
AMBupre-4	2	8	80	90	178	180
AMBupre-5	2	9	90	40	139	140
AMBupre-6	2	8	70	70	148	150
AMBupre-7	2	8	80	80	168	170
AMBupre-8	2	9	70	60	139	140
AMBupre-9	2	9	100	40	149	150
AMBupre-10	2	9	90	50	149	150
AMBupre-11	2	9	70	60	139	140
AMBupre-12	2	9	70	70	149	150
Median	1.6	8.4	80.0	55.0	143.3	145.0
Q1	1.5	8.0	70.0	40.0	130.6	132.5
Q3	2.0	8.5	90.0	70.0	148.5	150.0
Interquartille	0.5	0.5	20.0	30.0	17.9	17.5

With myocardial infarction surgery						
id souris :	Loss righing	induction	surgery stage	recovery	immobilization	return of righting reflex
MAMBupre+S-1	5	5	100	80	185	190
MAMBupre+S-2	5	5	90	90	185	190
MAMBupre+S-3	2	9	90	60	159	160
MAMBupre+S-4	2	8	90	70	168	170
MAMBupre+S-5	2	9	80	60	149	150
MAMBupre+S-6	2	9	90	50	159	150
MAMBupre+S-7	2	9	70	80	159	160
MAMBupre+S-8	2	9	70	90	169	170
MAMBupre+S-9	2	9	70	70	149	150
MAMBupre+S-10	2	9	70	70	149	150
Median	1.5	8.5	85.0	70.0	158.5	160.0
Q1	1.5	7.3	70.0	60.0	149.0	150.0
Q3	2.8	8.5	90.0	82.5	172.6	175.0
Interquartille	1.3	1.3	20;0	22.5	24.1	25.0
