# Supplementary material for: Evaluation of general anesthesia protocols for a highly controlled cardiac ischemia-reperfusion model in mice
Source: PLoS One. 2024 Oct 25;19(10):e0309799. doi: 10.1371/journal.pone.0309799 (PMC11508169; doi:10.1371/journal.pone.0309799)
Supplement: S4 Fig — (PDF) [file pone.0309799.s004.pdf]

Without atipamezole						
id souris :	Loss of righting reflex	Induction	Surgery stage	recovery	Immobilization	return of righting reflex
AMBupre-1	2	8	90	20	118	120
AMBupre-2	2	8	80	40	128	130
AMBupre-3	2	8	80	40	128	130
AMBupre-4	2	8	80	90	178	180
AMBupre-5	2	9	90	40	139	140
AMBupre-6	2	8	70	70	148	150
AMBupre-7	2	8	80	80	168	170
AMBupre-8	2	9	70	60	139	140
AMBupre-9	2	9	100	40	149	150
AMBupre-10	2	9	90	50	149	150
AMBupre-11	2	9	70	60	139	140
AMBupre-12	2	9	70	70	149	150
Median	1.6	8.4	80.0	55.0	143.3	145.0
Q1	1.5	8.0	70.0	40.0	130.6	132.5
Q3	2.0	8.5	90.0	70.0	148.5	150.0
Interquartille	0.5	0.5	20.0	30.0	17.9	17.5

With atipamezole						
id souris :	Loss righing	induction	surgery stage	recovery	immobilization	return of righting reflex
AMBupre+A-1	2	8	70	10	88	90
AMBupre+A-2	2	9	60	20	89	90
AMBupre+A-3	2	9	70	20	99	100
AMBupre+A-4	2	9	70	20	99	100
AMBupre+A-5	2	8	60	40	108	110
AMBupre+A-6	2	8	70	20	98	100
AMBupre+A-7	2	8	60	20	88	90
AMBupre+A-8	2	8	60	30	98	100
AMBupre+A-9	2	8	60	30	98	100
AMBupre+A-10	2	9	70	10	89	90
Median	1.9	8.1	65.0	20.0	98.0	100.0
Q1	1.5	8.0	60.0	17.5	88.4	90.0
Q3	2.0	8.5	70.0	30.0	98.6	100.0
Interquartille	0.5	0.5	10.0	12.5	10.2	10.0
