# Supplementary material for: Evaluation of general anesthesia protocols for a highly controlled cardiac ischemia-reperfusion model in mice
Source: PLoS One. 2024 Oct 25;19(10):e0309799. doi: 10.1371/journal.pone.0309799 (PMC11508169; doi:10.1371/journal.pone.0309799)
Supplement: S5 Fig — (PDF) [file pone.0309799.s005.pdf]

Alfaxan/Medetomidine/Buprenorphine	AMBupre+S-7	AMBupre+S-1	AMBupre+S-2	AMBupre+S-3	AMBupre+S-4	AMBupre+S-5	AMBupre+S-6	AMBupre+S-8	AMBupre+S-9	AMBupre+S-10
Body Weight	29	30.7	27	27.5	25.2	22.8	22.7	26.2	28	26.8
LV weight	75.6	59.22	58.45	78.8	81.9	73.8	73.9	69.8	79.8	81.1
AAR	30.0	28.7	28.1	28.0	27.9	38.1	29.3	26.7	26.0	31.4
AN	15.7	18.8	16.9	14.3	14.5	24.5	20.1	15.5	16.5	16.7
AAR/LV	40%	48%	48%	36%	34%	44%	40%	31%	33%	39%
AN/LV	21%	32%	29%	18%	18%	28%	27%	18%	21%	21%
AN/AAR	52%	65%	60%	51%	52%	64%	69%	58%	63%	53%

Alfaxan/Medetomidine/Buprenorphine + Atipamezole	AMBupre+S+A-1	AMBupre+S+A-2	AMBupre+S+A-3	AMBupre+S+A-4	AMBupre+S+A-5	AMBupre+S+A-6	AMBupre+S+A-7	AMBupre+S+A-8	AMBupre+S+A-9	AMBupre+S+A-10
Body Weight	27.2	25.4	24.9	25.7	26.4	26.2	26.9	23.1	24.2	25.2
LV weight	66.6	68.7	59.1	80.6	68.7	71.8	64	58.7	66.4	69.7
AAR	21.9	29.5	20.2	33.9	32.2	29.8	24.4	21.1	27.9	26.1
AN	11.2	18.1	10.9	21.6	19.2	16.7	15.6	11.2	15.3	11.3
AAR/LV	33%	43%	34%	42%	47%	41%	38%	36%	42%	37%
AN/LV	17%	26%	19%	27%	28%	23%	24%	19%	23%	16%
AN/AAR	51%	61%	54%	64%	60%	56%	64%	53%	55%	43%
