# Supplementary material for: Evaluation of general anesthesia protocols for a highly controlled cardiac ischemia-reperfusion model in mice
Source: PLoS One. 2024 Oct 25;19(10):e0309799. doi: 10.1371/journal.pone.0309799 (PMC11508169; doi:10.1371/journal.pone.0309799)
Supplement: S1 Table — (PDF) [file pone.0309799.s006.pdf]

Non invasive blood pressure

A/Me/Bp without surgery	AMBupre-1	AMBupre-2	AMBupre-3	AMBupre-4	AMBupre-5	AMBupre-6	AMBupre-7	AMBupre-8	AMBupre-9	AMBupre-10	AMBupre-11	AMBupre-12
10 minutes of anesthesia	77	103				104	79	99	90	67	70	159
(ET)	12	8				1	10	8	4	2	10	12
During ischemia	68	72				87	104	99	65	67	68	116
(ET)	3	5				3	7	5	8	3	11	12
After reperfusion	76	67				91	124	107	59	73	90	116
(ET)	2	1				2	7	4	1	1	1	6
	10 min b. I	during I	After R									
Mean	94	83	89									
ET	28	19	23									

A/Me/Bp with surgery	AMBupre+S-1	AMBupre+S-2	AMBupre+S-3	AMBupre+S-4	AMBupre+S-5	AMBupre+S-6	AMBupre+S-7	AMBupre+S-8	AMBupre+S-9	AMBupre+S-10
10 minutes of anesthesia		98	77	74	119	109	159	82	81	140
(ET)		14	1	0	24	5	12	32	0	8
During ischemia		70	72	64	95	59	116	80	75	104
(ET)		7	12	7	5	9	12	9	9	18
After reperfusion		56	63	64	82	64	116	73	75	95
(ET)		3	3	1	12	1	6	7	3	0
	10 min b. I	during I	After R							
Mean	104	82	76							
ET	30	19	19							

Heart Rate

A/Me/Bp without surgery	AMBupre-1	AMBupre-2	AMBupre-3	AMBupre-4	AMBupre-5	AMBupre-6	AMBupre-7	AMBupre-8	AMBupre-9	AMBupre-10	AMBupre-11	AMBupre-12
10 minutes of anesthesia	348	406	404	373	305	427	353	493	352	346	394	357
(ET)	5	6	109	34	119	55	11	14	39	3	60	3
During ischemia	333	345	350	292	326	462	333	459	347	326	360	286
(ET)	6	14	127	13	15	53	19	60	13	7	57	17
After reperfusion	340	344	377	291	336	430	316		336	331	307	267
(ET)	3	2	66	5	9	47	4		1	4	24	3
	10 min b. I	during I	After R									
Mean	380	352	334									
ET	49	55	43									

A/Me/Bp with surgery	AMBupre+S-1	AMBupre+S-2	AMBupre+S-3	AMBupre+S-4	AMBupre+S-5	AMBupre+S-6	AMBupre+S-7	AMBupre+S-8	AMBupre+S-9	AMBupre+S-10
10 minutes of anesthesia			462	334	298	307	329	363	411	
(ET)			39	55	13	56	76	6	13	
During ischemia	331	306	239	277		350	287	312	330	244
(ET)	27	32	7	30		29	79	16	36	24
After reperfusion	299		218			341	295	299	261	225
(ET)	25		31			31	81	54	76	50
	10 min b. I	during I	After R							
Mean	358	297	277							
ET	60	39	44							

Temperatures

	A/Me/Bp without surgery	AMBupre-1	AMBupre-2	AMBupre-3	AMBupre-4	AMBupre-5	AMBupre-6	AMBupre-7	AMBupre-8	AMBupre-9	AMBupre-10	AMBupre-11	AMBupre-12
PAD	10 minutes of anesthesia	36.2	37.1	42.0	41.6	37.4	31.6	38.8	33.6	40.4	36.9	37.5	41.0
	(ET)	2.1	1.8	0.2	0.8	2.4	2.0	0.3	1.3	0.6	1.1	0.8	0.7
	During ischemia	35.3	35.8	38.9	36.7	34.3	33.5	36.6	34.2	40.5	34.5	35.0	37.7
	(ET)	1.7	1.1	1.7	2.2	2.0	1.8	1.7	2.0	0.3	2.0	1.0	1.7
	After reperfusion	34.6	35.6	39.7	34.1	32.4	32.5	36.3	33.7	40.4	34.6	34.7	37.1
	(ET)	1.3	0.8	0.6	1.2	0.7	1.6	1.4	1.7	0.1	1.7	0.9	1.5
Body	10 minutes of anesthesia	37.5	37.4	34.4	37.3	37.7	37.5	36.6	37.4	36.9	37.2	37.4	37.5
	(ET)	0.1	0.2	0.3	0.3	0.2	0.1	0.5	0.2	0.1	0.1	0.3	0.2
	During ischemia	37.2	37.3	37.0	37.2	37.3	37.2	37.2	37.3	37.3	37.3	37.2	37.2
	(ET)	0.2	0.2	0.4	0.3	0.4	0.3	0.4	0.4	0.1	0.3	0.2	0.3
	After reperfusion	37.3	37.3	36.9	37.3	37.2	37.5	37.0	37.8	37.3	37.7	37.1	36.9
	(ET)	0.2	0.2	0.4	0.3	0.3	0.2	0.2	0.2	0.1	0.2	0.1	0.2
		10 min b. I		during I		After R							
		Pad temp	Body temp	Pad temp	Body temp		Pad temp	Body temp					
	Mean	37.8	37.1	36.0	37.2		35.4	37.3					
	ET	3.2	0.9	2.2	0.1		2.7	0.3					

	A/Me/Bp with surgery	AMBupre+S-1	AMBupre+S-2	AMBupre+S-3	AMBupre+S-4	AMBupre+S-5	AMBupre+S-6	AMBupre+S-7	AMBupre+S-8	AMBupre+S-9	AMBupre+S-10
PAD	10 minutes of anesthesia	38.1	39.4	39.2	39.4	36.5	38.1	35.4	40.7		41.4
	(ET)	0.6	0.5	0.2	0.3	1.9	2.7	0.9	0.2		0.1
	During ischemia	39.5	40.0	37.4	35.8	36.5	36.5	39.4	38.7	36.6	35.9
	(ET)	0.5	0.7	0.9	2.1	0.6	2.2	0.6	1.2	1.5	1.9
	After reperfusion	38.5	40.3	36.3	31.7	35.8	37.3	37.3	37.5	35.4	37.1
	(ET)	0.1	0.2	0.5	1.3	0.1	0.7	1.5	1.0	0.6	1.1
Body	10 minutes of anesthesia	35.7	36.3	36.7	36.1	37.3	37.5	37.3	36.7		36.5
	(ET)	0.1	0.1	0.2	0.2	0.2	0.3	0.1	0.2		0.1
	During ischemia	36.7	37.0	37.1	37.2	37.3	37.1	37.1	37.0	37.2	37.3
	(ET)	0.3	0.1	0.2	0.3	0.1	0.3	0.1	0.2	0.2	0.2
	After reperfusion	37.0	37.1	37.4	37.4	37.4	37.3	37.5	37.4	37.4	37.2
	(ET)	0.1	0.0	0.1	0.2	0.1	0.3	0.1	0.1	0.1	0.3
		10 min b. I		during I		After R					
		Pad temp	Body temp	Pad temp	Body temp		Pad temp	Body temp			
	Mean	38.7	36.7	37.6	37.1		36.7	37.3			
	ET	1.9	0.6	1.6	0.2		2.2	0.1			

EtCO2

A/Me/Bp with surgery	AMBupre+S-1	AMBupre+S-2	AMBupre+S-3	AMBupre+S-4	AMBupre+S-5	AMBupre+S-6	AMBupre+S-7	AMBupre+S-8	AMBupre+S-9	AMBupre+S-10
10 minutes of anesthesia	15	16	18	17	17	10	18	21		17
(ET)	5	7	5	5	6	6	5	9		7
During ischemia	19	19	16	17	15	14	18	18	29	19
(ET)	1	1	1	1	1	1	1	1	4	2
After reperfusion	18	17	16	16	14	14	18	17	19	21
(ET)	0	1	1	1	1	1	0	0	0	6

	10 min b. I	during I	After R
Mean	17	18	17
ET	3	4	2
