# Supplementary material for: Evaluation of general anesthesia protocols for a highly controlled cardiac ischemia-reperfusion model in mice
Source: PLoS One. 2024 Oct 25;19(10):e0309799. doi: 10.1371/journal.pone.0309799 (PMC11508169; doi:10.1371/journal.pone.0309799)
Supplement: Attachment — Submitted filename: Response to Reviewers.docx [file pone.0309799.s007.docx]

**Response to Reviewers**

First, we would like to thank the reviewers for reviewing our paper. Thanks for the comments, tips and corrections. We have modified the manuscript and figures/tables as required and hope to have answered to all questions.

Answers to raised points :

**Reviewer #1**

**1- Could you explain the reason for selecting buprenorphine and butorphanol as analgesics? Were there any specific considerations regarding their efficacy or potential side effects?**

We tested butorphanol and buprenorphine with the advice of veterinaries, because both opiods are commonly used in surgery procedures. We finally kept Burenorphine 1) as there was no difference between the two analgesics on anesthesia times in our conditions and 2) because buprenorphine has a longer time of action (estimated at 6 to 12 hours) than butorphanol (estimated at 2 to 4 hours) faciliting the maintenance of postoperative analgesia. Moreover, it has been showed that k-opioid receptor activation is involved in myocardial infarction while the role of mu-opioid receptor seems less clear, suggesting that buprenorphine would induce less bias in our model than butorphanol.

**2- Concerning the anesthetic protocol, choosing to antagonize the medetomidine action is relevant and improves the recovery of the animal. However, as analgesia is supported by medetomidine (central analgesia) and butorphanol (slight visceral analgesia), the reversal of the action of medetomidine abolishes its analgesic action and exposes the rat to pain. Why the authors do not use local anesthetics such as lidocaine that would bring a strong peripheral analgesia? Moreover, postoperative analgesia is very light and no data are given concerning the presence and intensity of pain during the postoperative period. The authors have to present data concerning pain assessment in the animals models.**

- We fully share this analysis concerning antagonization of medetomidine. Local anesthesic was administred but was not mentioned in the manuscript (now Corrected).
 - Post-operative pain have been evaluated in all operated mice and corresponding informations have now been added in methods and results sections.

**3- Did you observe any differences in post-operative pain scores or recovery profiles among the experimental groups?**

Post-operative scores (evaluate at 1-2h after waking up) were similar for all mice, wathever the anesthetic protocol : This Score was 1, due to "hypoactivity, reduced mobility" attributed to residual anesthesics. Activity of mice normalized after a few hours. No other clinical signs in relation to the established score grid (particulary related to animal behaviour or breathing) were identified.

**4- Are there any insights into the underlying pharmacological mechanisms that might explain why the alfaxalone/medetomidine/buprenorphine protocol was superior for mice cardiac surgery? For example, inflammation that could be relevant to ischemia-reperfusion injury.**

We showed that the Alfaxalone/medetomidine/buprenorphine protocol is superior in terms of compatibility with the surgical model: duration and depth of anesthesia adapted to cardiac surgery, possibility of reversal. However, it does not seem superior in terms of ischemia-reperfusion injury : no benefit on the size of infarction (.e. cardioprotection) was highlighted in our conditions.

**5- In the manuscript and title, both 'myocardial infarction' and 'ischemia-reperfusion model' are used to refer to the experimental model. To ensure consistency throughout the text, would it be appropriate to standardize the terminology by selecting one term to describe the model? (ischemia-reperfusion model)**

The text has been standardized using the term 'cardiac ischemia-reperfusion' to characterize the model.

**6- Could the authors consider including representative images of triphenyltetrazolium chloride (TTC) staining from each experimental group to visually demonstrate the evaluation of infarct size in the operated mice? The addition of such images would enhance the clarity of the results section.**

Representative Images were added in Figure 1 to illustrate the results

**7- Has the potential impact of the anesthetic protocols on respiratory function been considered, especially given that certain anesthetic agents utilized in the study have been associated with respiratory depression?**

Impact on respiratory function was not specifically investigated in this study. However, several cases of respiratory depression leading to death were reported in our previous experiments with 21%O2 supply during anesthesia. In this study, oxygen delivery has therefore been increased to 30% and no more respiratory depression were observed in this condition exept for alfaxalone:midazolam+buprenorphine group that have therefore been stopped.

**8- The general conclusion and evaluation methods used in the present study appear to closely resemble those described in a previously published article by Higuchi et al. ('Evaluation of a combination of alfaxalone with medetomidine and butorphanol for inducing surgical anesthesia in laboratory mice,' Japanese Journal of Veterinary Research, 2016). Could the authors provide clarification and about the novelty of the current research.**

We have chosen to evaluate the time-related parameters of anesthesia with the most widely used method in literature, including publication of Higushi et al, in order to facilitate comparison of results.
Higuchi et al. compared different dosages of alfaxalone and medetomidine and different administration routes to propose an a priori suitable anesthesia protocol for "surgery". The main novelties of the current research were therefore 1) to compared this protocol to multiple other anesthetic protocols used in mice, 2) to show that butorphanol can be replaced by another opioid whose duration of action is longer, without negative impact on anesthesia parameters and 3) to show that the selected protocol is specificaly suitable to thoracic surgery and cardiac ischemia-reperfusion model, with no impact of atipamezole on measured infarction size.

**Reviewer #2**

**Major comments :**

**- Line 72. What is meant by “ immobilization time”?**

Immobilization time is defined as the time span between the loss and recovery of righting reflex. The definition was added in the manuscript.

**- Line 97. What is reported is certainly true, but the rabbit is not a rodent and the focus of the study seems to be on rodents, particularly mice. Perhaps it would be more appropriate to speak generically about the cardiovascular effects of drugs without referring to a particular species, or referring specifically to mice, since the response to drugs can vary greatly depending on the species.**

The sentence has been modified accordingly.

**- Line 119. Add references for the studies cited on the role of estrogens.**

References have been added

**- Lines 132-134. So, how many animals were needed? How many were involved and in which groups? It should already be reported here, even if explained in detail later. Furthermore, it is not further specified how the use of butorphanol and buprenorphine was chosen, because from Figure 1 it seems that all animals received butorphanol . The groups should each be indicated with an acronym for clarity, and with the number of animals for each group.**

The number of animals per group has been detailed in the "animal model and surgery" paragraph.
Because of ethical considerations, it was not envisaged to test butorphanol AND buprenorphine with all anesthesia protocols. We so have decided to first test every protocols with one of the opioid (butorphanol) then test 2 selected protocols with the second opioid (buprenorphine).
The use of acronyms (like "AMeBt") could indeed facilitate the understanding of the Figure 1 results (comparison of 6 different protocols) but would complicate the understanding of the rest of the results (generally comparison of two conditions with the same anesthesia protocol)

**- Line 136. This entire section is unclear and difficult to repeat. The administration areas, containment, type of gaseous circuit used, etc... are missing.**

The section has been amended.

**- Line 137. How were the drugs diluted? It would be useful for the reader who wants to replicate the procedure.**

A column "dilution" has been added to table 1

**- Table 1. The dose of atipamezole is the same as that of medetomidine. Why this choice? Normally the dose of atipamezole is 2.5-5 times that of medetomidine .**

This question was not specifically assesed in our paper but dose of atipamezole is particularly depending on the animal species (for example, the drug manufacturers generally advise doses 5 times that of medetomidine in dog and 2,5 times in cat) and timing of injection after anesthesia induction (for example, when administred 30 minutes after anesthesia in mice, doses of 0,3 and 1,5mg/kg of atipamezole worked equally : Kirihara et al. 2015). Furthermore, the dose of atipamezole we selected here (0,3mg/kg, for 0,3mg/kg of Medetomidine) was commonly used. However, a recent publication (Tashiro et al. 2022) shows that larger doses of atipemazole could have benefits, particularly on mice temperature. A paragraph has been added in discussion to adress this point.

**- Table 1. Why were ketamine and xylazine injected intraperitoneally, while all other drugs were injected subcutaneously? Ketamine and xylazine also have good subcutaneous absorption.**

Since ketamine and xylazine were still commonly used with intraperitoneal injections, that is why we maintained this administration route. However, more and more studies are using subcutaneous injection for both ketamine and xylazine so it would now be interesting to also test this refined injection route.

**- Line 140-141. Here too, it would be useful to add a reference to justify this concept.**

References have been added

**- Line 143. How did the physical restraint for the injection occur? Were special tubes used? In which area was the subcutaneous injection performed?**

The mouse is held in the hand and the subcutaneous injection was made in the lower right dial of the abdomen. Information added in methods section

**- Line 146. Which gas circuit was used? Was a mask used? Were they intubated? If yes, with what procedure?**

The gas circuit is composed of air and oxygen bottles connected to a adjustable gaz mixer and a flowmeter allowing to deliver 30% oxygen to the animal via a mask. Informations added in methods section.

**- Line 147. So, the non-reversal group underwent the procedures before the others? Also, which group did not receive atipamezole ?**

Indeed, the non-reversal group underwent the procedures before the groups with reversal : After selecting the best anesthetic protocol, two groups received atipamezole injection, with or without surgery, to study the impact of atipamezole on measured anesthesia parameters and infarct size. Clarifications on chronology and groups have been made in the manuscript.

**- Line 152 and following. I think the city of the manufacturer is also necessary .**

It seems that this requirement is not mentionned in Plos One Guidelines to authors and that state is generally sufficient in publications. To lighten the paragraph, we therefore prefer to mention only the city

**- Line 168. These are not anesthesia parameters but more than anything else they are the evaluation of some reflexes. I would suggest changing the paragraph title.**

The title has been replaced by: "Depth of anesthesia evaluation by scoring of reflexes"

**- Table 2. How was the air blown on the mice eyes to test the reflex?**

The ocular reflex was evaluated by blowing air, through a 5 ml syringe, on the cornea, and observing the presence (score=0) or absence (score=1) of a reflex. Information added to table 2

**- Table 2. What is meant by “time” in the table, when evaluating reflexes? Do you intend to talk about the anesthesia plan? Time is a word that may not be clear in this context.**

 The term "Surgical time" has been replaced by "Surgical anesthesia time".
 The precise definition of the different times-related parameters of anesthesia has been added : Immobilization time is defined as the time span between the loss and recovery of righting reflex. Induction time is defined as the time span between loss of right reflex and the start of surgical anesthesia. Surgical anesthesia time is defined as the time during which the score is maximum. Recovery time is defined as the time from the end of surgical anesthesia until the righting reflex returns (Table 2).

**- Line 177. Here is a repetition of what was said before. I suggest reporting the information in just one point, and possibly specifying in which paragraph it will be found.**

This suggestion has been taken into account in the correction,

**- Line 177. How were they intubated? With what technique and what tubes? What ventilation settings (pressure, acts, etc.)?**

The metal intubation tube was inserded in the mouse's mouth and connected to the Physiosuite ventilator (Kent scientific, USA) whose breath rate and tidal volume are automatically adapted to the weight of animal. We only added essential informations in the manuscript because we think that intubation technique does not affect the results of measured parameters in this study.

**- Line 179. How were these parameters monitored? With what tools?**

Body temperature, pad temperature and end-tidal carbon dioxide (EtCO2) were continuously monitored thanks to sensors (temperature probes and capnograph) connected to the Physiosuite ventilator

**- Line 179. It is not the system that is placed on the tail, but the sleeve. The sentence should be written better.**

Corrected

**- Line 180. What is meant by “ immobilization time”? when using these terms, specific definitions should be given to rely on.**

Immobilization time is defined as the time span between the loss and recovery of righting reflex. Precise definitions of all time-related parameters added in the article.

**- Line 181. Better indicate the area where the needles were inserted and how they were connected to the ECG system. Indicate the name of the monitoring system. Also, “on” is not appropriate if the insertion of an instrument into the subcutaneous tissue is involved.**

Subcutaneous needles of electrocardiogram (ECG) monitoring system (Emka, Paris, France) were placed at the right forearm, right leg and left leg.

**- Line 185. What suture pattern?**

The suture pattern is simple overlock

**- Line 188. Indicate the name and details of the manufacturer for the incubator.**

Informations added to the methods section

**- Line 188. What is meant by “ total recovery”? give a definition. Furthermore, following the meaning of the sentence, we understand that the food and water were left only until recovery, and then removed. Is that so?**

Total recovery means recovery of all normal behaviors (alimentation, movments, etc.) and normal reactions to stimuli of environment. Upon waking, gelled food and gelled water are given to the animal to facilitate food intake in addition of usual food and bottle of water. Informations added in methods section

**- Lines 189. Although the focus is on anesthesia, the methods should also be briefly described here.**

Detailed method added in the manuscript

**- Line 193. Since nonparametric tests were performed, it is assumed that the distribution was not considered normal based on the Shapiro-Wilk test. Therefore, the use of median and interquartile ranges for describing data would be more appropriate than the use of mean and SD.**

When appropriate, mean+/ET has been replaced by mean+/-Interquartile ranges

**- Line 198. What “times” are you referring to? It has not been specified anywhere previously in the text, except perhaps in Table 2. However, Table 2 is not at all clear and there is no adequate definition of what is meant. Furthermore, the evaluation of reflexes can allow us to hypothesize a specific condition, rather than a "time". I believe that the reasoning behind this analysis should be explained better because it is not clear at the moment. Furthermore, in the "Statistical analysis " section it would be more appropriate to clearly talk about which tests were done on which parameters specifically.**

The precise definition of each related-time parameter of anesthesia has been added (parapgrah "Time-related parameters of anesthesia" in Methods)
 As mentioned in statistical paragraph, statistical test is the same for all parameters and, for each parameter, all protocols are paired-wise compared

**- Line 210. From Figure 1 it is not at all clear that LRR was lost in less than 3 minutes, being a figure with a Y axis ranging from 0 to 200. The 3 minute point is imperceptible. I suggest modifying the figure, and possibly separating it.**

The main purpose of this graph was to primarily highlight the impact of time-related parameters compared, each to each other and each to immobilization time, in the various groups. That is why it was important to us to chose an unique scale. In this way, for example, one can quickly visualize that recovery time is substantial compared to surgery time in Protocol 2, but not in protocol 3. On the other han, This choice effectively implies not being able to precisly visualize duration of some parameters, that's why the most relevant values and differences were detailed in the text. Moreover, even if there are several statistical differences in LRR measurement between protocols , the only interesting information concerning this related-time parameter of anesthesia remains for us that the loss of righting reflex is very quick in all protocol, allowing the experimenter to quickly repare the mice for surgery after anesthsia induction.

**- Line 215. So, in these 2 animals, the procedure was not performed?**

These animals followed the anesthesia procedure but never exceeded the score of 4 so they did not reach the stage of 5 (= surgery anesthesia). No surgery procedure was made with this anesthesia protocol in the study

**- Line 220-221. So, was the duration of surgical anesthesia compared with statistical tests? This must be specified in the statistical analysis part which, as mentioned, is not clear.**

As mentioned in statistical paragraph (in Methods), statistical test is the same for all parameters and, for each parameter (including time-related parameter "surgery stage" ), all protocols are paired-wise compared. The higher values for this time-related parameter (i.e. "surgery stage") were observed in Protocol 3 and Protocol 4 and it even was significantly higher in Protocol 3, compared to Protocols 1, 2 and 5.

**- Line 223. According to what was written before, the animals had been intubated. why are we talking about face masks here? Also, which mask? What type of ventilation?**

Only the animals that undergo the surgical procedure are intubated the others are under mask. The material and method have been modified for clarity.

**- Line 224. On what basis is it presumed to be “ potentially due to cardiorespiratory failures ”? Was there apnea? How did the heart rate change? What was done with the 2 untested mice, given that we stopped at 4? The total number declared in the abstract is 72. The number is not included in the text, and this is lacking information.**

Indeed, we observed apnea before cardiac arrest and death (but we did not measure ECG on these groups) suggesting cardiorespiratory failures.
 Many in vivo projects are carried out in our animal facility on C57/bl6J mice. Animals that have been ordered for this project but not used for it, have finaly been used in another project of the laboratory.
 72 animals were used for the comparison of the 6 different anesthesia protocols but 60 mice are presented in the figure 1 (with 30% O2 supply in 5 full studied protocols) and 12 mice have been excluded of analysis (8 mice, because they were supplied with 21% O2, and 4 mice because they were involved in the abandonned protocol (i.e. Alfaxalone+Midazolam+Butorphanol)). However, the wording can indeed be misleading, so we have clarified the numbers of animals by indicating in abstract only those submitted to anesthesia protocols with 30% O2.

**- Line 228. According to the reported route of administration, ketamine is administered intraperitoneally while medetomidine is administered subcutaneously. Therefore, it is not a “single injection”.**

This mistake has been Corrected

**- Line 231. Since ketamine , medetomidine and xylazine also have analgesic power, unlike alfaxalone and midazolam , the role of analgesia is already partly important in the evaluation of protocols without the use of opioids. Thus, the role of analgesia was not evaluated, but the role of adding an opioid to the protocol. I suggest changing the title of the paragraph and considering this aspect in the discussion.**

Text and title have been changed and the information has been added to the discussion

**- Line 233. From Figure 1, it appears that all animals in the previous evaluation received butorphanol , but this was never specified before. Is this an error in the table? However, if it has been administered to everyone, the combination of drugs itself must be analyzed considering the effects of the opioid.**

Text has been changed for clarity

**- Line 234 and following. Was this assessment done in new groups? Or are they the same groups we were talking about before? If they are new groups, specify. On how many animals was the procedure carried out? However, if we are talking about animals in the groups previously described, the use of opioids certainly changes the assessment made. It is not clear which animals and which groups we are talking about, and whether these evaluations were carried out only after the first analyzes had already been carried out, on the protocols considered to be the best.**

The butorphanol groups in Figure 3 are the same as those in Figure 2. Conditions were the same in all compared groups (exept studied variable : anesthetic protocol in fig2, opioid in figure3) and statistics take these considerations into account. We have clarified the different groups number and the timing in the manuscript.

**- Line 236. How were the comparisons made? With what test? Are there any significant results or not? If a statistical analysis has not been performed, I believe it should be implemented instead.**

For each time-related parameter of anesthesia, Protocol 3 + buprenorphine was compared to Protocol 3 + butorphanol and Protocol 4 + buprenorphine was compared to Protocol 4 + butorphanol. As mentioned in statistical paragraph (in Methods), comparisons were made with Wilcoxon test. Pertinent results were detailed in next paragraph

**- Line 243. How time is determined is “ significantly higher ”? indicate a p value .**

P-value has been added.

**- Line 250. So, the procedure was performed in this group only? If so, the paragraph at line 174 should be partially moved here. Why was it placed before?**

Surgery procedure for infarction induction (as described in Mehods) was perfomed in two groups (i.e. "Protocol 4 + Buprenorphine + surgery" and "Protocol 4 + Buprenorphine + surgery + atipamezole") described in two distinct paragraphs of results section (i.e. "validation of alfaxalone/medetomidine protocol in a mouse model of cardiac ischemia-reperfusion" and "Side effet of the general anesthesia protocols on the infarction size".

**- Line 259. Is the EtCO2 value reported in the other groups? In what table or figure? What does figure 2C have to do with it?**

Indeed, mention of the figure 2C here was a mistake : It was "Table 3" (Corrected)

**- Line 261. Normally, " immobilization " means a phase in which the animal is open to manipulation, immediately after the administration of anesthetics. What is meant by “ immobilization ” in the context of the recovery phase? It seems counterintuitive.**

Immobilization time is defined as the time span between the loss and recovery of righting reflex.this corresponds to the time during which the animal is motionless. Recovery time is defined as the time from the end of surgical stage until the righting reflex returns. Immobilization time is the sum of induction time, surgical anesthesia time and recovery time. The definitions have been added to the methods section.

**- Line 263-264. We talk about animals that have undergone surgery and animals that have not undergone it, and then 3 differences in times are reported. It is not clear which group is being referred to. Indicate.**

The groups have been specified in the text.

**- Line 266. Systolic blood pressure.**

Initial values reported in manuscript were "mean pressures". Systolic pressure is now reported in manuscript instead of mean pressure (results and statistic have been updated)

**- Table 3. The table description is confusing. The table should be set up differently, in order to insert the IP value of the comparisons made, so that they are clear.**

Table, table legend and text have been modified to explain more what is compared : 1) Global values and evolution of parameters are discussed and 2) for each parameter, what that is the difference between timepoint values inside a given group that was statisticaly analyzed.

**- Table 3. Why EtCO2 is expressed as “ arbitrary unit ”? normally we talk about mmHg.**

Indeed, values obtained are expressed in mmHg. However, because of the high respiratory frequency, low tidal volume and the distance between capnograph and mouse, the values obtained with the capnograph in mouse is underestimated and cannot be considered as an absolute value. That is why we first have chosen to expressed EtCO2 like an arbitrary unit. the only information we analyzed on EtCO2 is so it's variation. Anyway, Unit (for mmHg) has been changed.

**- Line 289. Body temperature was maintained, but there was a difference in pad temperature . Why? Since the parameter has been recorded, it would be appropriate to discuss it briefly.**

Informations now added in results sections and discussed in discussion section.

**- Lines 289-290. EtCO2 certainly reflects blood pCO2, but its variation can depend on various factors. Furthermore, the reported values are rather low to be considered normal: again, the monitoring system can influence this analysis, as the tidal volume is very low in this species. However, these aspects require a brief discussion. Furthermore, during the ischemia phase there was an increase, as expected.**

These points are now discussed in discussion section. Increased EtCO2 during thoracic opening would indeed be coherent but this increase was not significant in our conditions.

**- Line 295. Here too, it is not well defined how many subjects were tested, on which groups, etc.**

Groups have been detailed and Flow chart has been added in supplemental data

**- Line 297. Is the recovery time assessment based on what is reported in Table 2? So, was it enough to obtain a score between 4 and 1 to consider the animal awake? It is a very wide range, which can be due to several factors. Not very indicative of an awakening. Does it mean the recovery of the righting reflex instead?**

Effectively the definition of recovery time has been added in the material and method. Recovery time is defined as the time from the end of surgical stage until the righting reflex returns. It is therefore the recovery of the righting reflex that is faster with the injection of atipamezole.

**- atipamezole was not administered , from what moment was the evaluation carried out? In these cases, time should be considered from a specific point valid for both groups.**

Recovery time being the time from the end of surgical stage until the righting reflex returns, it is valid for both groups. The definition of recovery time has been added in the material and method.

**- Lines 300-301. Were there cases in which monitoring was not possible because they were already awake? How many?**

Yes, 2 mice (AMBupre+A-1 and AMBupre+A-10) had recovery time that was less than 10 minutes.

**- Line 308. This procedure and following evaluation and analysis need to be better described and supported by statistical analysis, and p values should be reported.**

Statistical paragraph (in Methods) has been improved for this point. There were no significant difference of the infarction size between the groups. p values were reported in the manuscript (graph and text) only when significative, so as not to overburden the document.

**- Line 333. It was never reported, in the Materials and methods section, the use of 21% oxygen, but only the use of 30% oxygen.**

Those animals are not included in this study but because we mentioned them in results section, an explanation about this condition has now been added in methods section.

**- Line 333-334. Intubation alone does not solve respiratory problems if controlled ventilation with a set percentage of oxygen is not carried out. The phrase is a bit misleading.**

The sentence has been Corrected : Animals were immediately intubated and ventilated with 30% O2 after the righting was lost in our LAD occlusion model to help avoid those respiratory depression problems.

**- Line 337. The duration of the procedure should be reported in the results.**

Duration has been added (about 70 minutes)

**- Line 351. If the doses are the same, why was this investigation necessary?**

In our study, we didn't investigate anesthesic doses but the adequation of such a anesthesic protocol with our cardiac ischemiea-reperfusion surgical model.

**- Line 354. The surgical plane of anesthesia can differ among species, so the evaluation of reflexes is not necessarily an indicator of surgical plane.**

The sentence has been changed

**- Line 365. It is stated the infarction size was similar to other studies, but no data is reported, so it is not useful for the reader.**

This information is important for this study. Compared Values have been added in results and discussion sections.

**- Line 369. Did you have 10% mortality? Where is this declared?**

Exact mortality was 9.1% (2/22) in this study. This data has been added in result section.

**- Line 370. Was a negative pressure instituted to resolve pneumothorax after the procedures? This must be discussed in the Materials and methods section.**

The air in the thoracic cavity is sucked through a catheter connected to a syringe during suturing to prevent pneumothorax. The information has been added to the material and method.

**- Line 372. Why should 70 minutes be considered as a late administration? I would remove this adjective.**

Actually the sentence may be awkward. The adjective was added to link to the upstream article (Baker et al, 2011) that used the term early for a 10-minute injection and late for a 40-minute injection. the adjective has been removed.

**- Line 373. “ less than 10 minutes”. In the results, you state that the recovery was 22 ± 9 min.**

It’s a mistake. Some mice wake up in less than 10 minutes after injection but on average in less than 30 minutes. The correction was made in the text.

**- Line 378 “ alfaxalone+medetomidine+buprenorphine (80/0,3/1 mg.kg -1 , sc )”. In Table 1, the dose of buprenorphine is 0.075 mg/kg. Also, this error is found in the abstract.**

Corrected

**- Line 379. It was never stated that a “variable dosage” was used. What is meant by this phrase? Why is it not reported in the materials and methods and in the results?**

In our study, only dosage at 0.3 mg.kg-1 was used, but some data in litterature suggest that dosage must higher when injection is early after anesthesia induction. However, the sentence "variable dosage depending on injection timing" was indeed mislead and has therefore been deleted.

**- Lines 377-380. In the materials and methods, it is stated that the evaluation of the surgical procedure was made on the protocol based on alfaxalone and medetomidine . Buprenorphine is not mentioned. While reading, there is confusion.**

Clarification added in paragraph "Anesthetic protocols"

**Minor comments :**

**- Throughout the text. “infarct” -> “infaction”**

Corrected

**- Line 34. I would change the running Head to: “Mice anesthesia for myocardial infaction model”, so that it is more specific.**

Head running title changed for "Mice anesthesia for cardiac ischemia-reperfusion model

**- Line 38. “alfaxalone” instead of “ alfaxan ”**

Corrected

**- Line 62. Better to replace “mouse” with “mice”.**

Corrected

**- Line 71. “ did not induce major change in” -> “did not alter”**

Corrected

**- Line 82. “provide” -> “ provides ”**

Corrected

**- Line 82. Remove “a”.**

Corrected

**- Line 108. Reversal could be a better term.**

Corrected

**- Line 110. Commas are not needed.**

Corrected

**- Line 124. “ was ” instead of “ were ”, in both cases.**

Corrected

**- Line 124. Remove the bracket.**

Corrected

**- Line 144. “placed in a dorsal recumbency”: remove “a”.**

Corrected

**- Line 146. Same as line 144.**

Corrected

**- Line 146. “was” instead of “were”.**

Corrected

**- Line 152. “ alcyon ” -> “ Alcyon ”.**

Corrected

**- Table 2. “ refex ” -> “reflex”**

Corrected

**- Line 178. Remove the comma after “body”.**

Corrected

**- Line 179. Non-invasive.**

Corrected

**- Line 182. “ achieved ” -> “ performed ”**

Corrected

**- Line 183. A dot is missing.**

Corrected

**- Line 184. “loosened” -> “loosening”.**

Corrected

**- Line 186. “was” instead of “were”.**

Corrected

**- Line 188. Add an article before “ heating incubator”.**

Corrected

**- Line 193. Results are expressed .**

Corrected

**- Line 218. Surgical instead of surgery.**

Corrected

**- Results section . Sometimes it is read “ mn ”, sometimes “min”. follow the journal’s style of abbreviations.**

Corrected

**- Line 229. “ adding ” -> “ subsequently administering ”**

Corrected

**- Line 230. “ awakening ” -> “recovery”**

Corrected

**- Line 274. Remove “ were ”.**

Corrected

**- Line 286. “did” instead of “is”.**

Corrected

**- Line 299. I suggest “reduced” instead of “fell”.**

Corrected

**- Line 301. Effects.**

Corrected

**- Line 302. Were observed.**

Corrected

**- Line 323. Led.**

Corrected

**- Line 330. “That” instead of “what”.**

Corrected

**- Line 333. A dot is missing.**

Corrected

**- Line 341. “ we can read” -> “it has been reported”.**

Corrected

**- Line 343. “ sleeping time” -> “duration of anesthesia”.**

Corrected

**- Line 344. Induces.**

Corrected

**- Line 351. I suggest “The quality of surgical anesthesia…”**

Corrected

**- Line 354. Surgical.**

Corrected

**- Line 357. Remove “;”.**

Corrected

**- Line 365. Was.**

Corrected

**- Line 368. Peri-.**

Corrected
